# Withametelin inhibits TGF-β induced Epithelial-to-Mesenchymal Transition and Programmed-Death Ligand-1 expression *in vitro*

**DOI:** 10.3389/fonc.2024.1435516

**Published:** 2024-07-15

**Authors:** Ashna Fathima, Mohammad Ali Farboodniay Jahromi, Sajeli A. Begum, Trinath Jamma

**Affiliations:** ^1^ Cell Signaling Laboratory, Department of Biological Sciences, Birla Institute of Technology & Science, Hyderabad, India; ^2^ Medicinal Plants Processing Research Center, Shiraz University of Medical Sciences, Shiraz, Iran; ^3^ Department of Pharmacy, Birla Institute of Technology & Science, Hyderabad, India

**Keywords:** withanolides, cancer, PD-L1, EMT, metastasis

## Abstract

Withanolides are a group of naturally occurring plant-based small molecules known for their wide range of host cellular functions. The anticancer potential of withanolides has been explored in varying cancer cell lines *in vitro*. Based on our prior studies, among the tested withanolides, withametelin (WM) has shown significant cytotoxicity with the highest efficacy on HCT-116 colon cancer cells (IC_50_ 0.719 ± 0.12μM). Treatment with WM reduced the TGF-β driven proliferation, colony-forming ability, migration, and invasiveness of HCT-116 cells *in vitro*. WM also downregulated the expression of mesenchymal markers such as N-CADHERIN, SNAIL, and SLUG in HCT-116 cells. At the molecular level, WM inhibited TGF-β induced phosphorylation of SMAD2/3 and reduced the expression of an immune checkpoint inhibitor programmed-death ligand-1 (PD-L1). Our study highlights the possible anticancer mechanisms of WM involving modulation of the TGF-β pathway and associated target gene expression, suggesting its potential utility in cancer therapy.

## 1 Introduction

Colon Adenocarcinoma (COAD) is a major form of Colorectal Cancer (CRC), the third most prevalent cancer worldwide, and the second most common reason for cancer-related death (1). More than 50% of new cases of CRC were reported in Asia, followed by Europe and North America, with an estimated number of patients predicted to increase from 1.88 million in 2020 to 2.94 million in 2040 (2). The conventional treatments focus on controlling the symptoms with the help of pharmacotherapy, which includes corticosteroids, aminosalicylates, immunomodulators, and biologics (3). However, surgery remains a necessary means of treatment in advanced CRC. In addition, current CRC medications are associated with infectious and neoplastic side effects, suggesting a need for highly effective drugs or combinations of therapy (4).

Naturally occurring plant compounds, such as carotenoids, flavonoids, terpenoids, etc., account for a significant proportion of approved anticancer drugs (5, 6). Some of the members of these families are extensively investigated for their ability to modulate cellular signaling pathways and regulate tumorigenesis. Withanolides are one such class of compounds that has been in therapeutic application in traditional Ayurvedic and Unani medicines for over 3000 years (7). Withanolides are known for their wide range of functions, including anti-inflammatory (8), sedative (9), diuretic (10), cytotoxic (11), antimicrobial (12), and immunomodulatory potential (13). Among various withanolides, withametelin (WM) remains less explored, with a proven impact on non-small cell lung cancer cells (14). Some studies have shown that WM also exhibits drug-like properties in regulating inflammation, analgesia, brain disorders, and cancer (15). However, the exact downstream mechanism potentiating WM’s anticancer property remains unknown.

The development and progression of CRC follow various molecular mechanisms (proliferation, invasion, advancement, or inhibition of apoptosis in CRC cells). One of these mechanisms also involves Transforming growth factor-β (TGF-β) signaling, playing a critical role in angiogenesis in tumor microenvironments and differentiation of epithelial cells (16). The paradoxical dynamic of TGF-β signaling has complicated the understanding of its role in cancer biology. However, studies have reported the progression of tumors in the colon upon disruption of TGF-β signaling (17). Mechanistically, CRC metastasis involves the detachment and infiltration of adjacent tissues by the cancer cells from the primary site of origin (18). The critical factors contributing to metastatic CRC (mCRC) are loss of Epithelial-Mesenchymal transition (EMT) regulation, increased angiogenesis, provoked stemness, and immunoregulation dictated by the microenvironment in CRC (19). TGF-β signaling contributes to such mCRC by promoting EMT (20).

Recent studies have brought forth the involvement of TGF-β in tumor immune evasion (21). Immune tolerance is one of the major challenges in cancer immunotherapy, and PD-L1 is another such molecule that can contribute to immune suppression (22). PD-L1 upregulation is found to be more common in mCRC (23), and PD-L1 expression assists tumor cells in evading immune surveillance by enhancing Treg function. PD-L1 expressed on tumor cells binds with the PD-1 receptor on activated T cells, inhibiting cytotoxic T cells (24). PD-L1 expression is significantly upregulated in tumor tissues via various mechanisms. Interestingly, a positive correlation between the expression of TGF-β and PD-L1 has been observed in CRC (24). This highlights the need for a dual-targeting strategy to regulate the inhibitory effect of immune checkpoints displayed by PD-L1 and TGF-β pathways in CRC.

The present study mainly focuses on exploring the impact of WM in regulating TGF-β signaling in mCRC. Human colon cancer cells (HCT-116) were treated with WM, and we observed a significant reduction in cell proliferation, colony-forming ability, and migration in a dose-dependent manner, indicating the anticancer potential of WM. Additionally, TGF-β induced EMT also reduced markedly upon treatment with WM. Further analysis of the downstream signaling highlighted that WM treatment inhibited phosphorylation of SMAD2/3 protein complex, which may have regulated the associated gene expression. Our study also highlighted the immune modulatory potential of WM, as it could inhibit the expression of PD-L1, which may eventually make the cancer cells more susceptible to immune cell-mediated cell death. Overall, our study suggests that the ability of WM to modulate TGF-β induced cancer progression and PD-L1 expression may contribute to it being a potential candidate for combinatorial therapy for mCRC.

## 2 Materials and methods

### 2.1 Cell culture

HCT-116 cells were purchased from Cell Repository NCCS, Pune, and cultured in complete DMEM (Thermo Scientific, Gibco, #12100061), supplemented with 10% heat-inactivated FBS (Thermo Scientific, Gibco Brazil, #10270106) and 1X PenStrep (Thermo Scientific, #15140122) in a humidified incubator at 37°C and 5% CO_2_.

### 2.2 Cytotoxicity

WM was isolated from the ethanol extract of *Datura innoxia* leaves through column chromatography. The detailed procedure for the isolation, purification, and characterization of WM has been presented in our earlier publication (14). MTT assay was performed to understand the effect of WM on cell viability and proliferation. HCT-116 cells were seeded at a concentration of 2000 cells/100μL medium/well in a 96-well plate; once adhered, they were treated with various concentrations of WM from 0-100μM. The cells were incubated for 72 hours, and the cell viability was analyzed using MTT according to the manufacturer’s instructions. Briefly, 10μL of MTT solution (5mg/ml) was added to each well and incubated for 4 h at 37°C. The formazan crystals formed were dissolved in 200μL of solubilization solution (DMSO), and absorbance was taken at 570 nm.

### 2.3 Colony forming

HCT-116 cells were seeded in a six-well culture plate at 500 cells/well density and treated with DMSO, 0.1μM, and 0.5μM WM in culture medium for 9 days. The colonies formed were fixed with a 3:1 ratio of methanol and glacial acetic acid for 10 min. The cells were then washed with 1X PBS and stained with 0.5% v/v crystal violet solution (dissolved in 25% methanol) for 10 min. Colonies were counted visually or using ImageJ software, NIH (Version 1.53K). The stain taken up by the colonies was dissolved in 10% SDS, and absorbance was recorded at 540 nm.

### 2.4 Wound healing

0.2X10^6^ HCT-116 cells were seeded in a 12-well tissue culture plate and incubated till a uniform monolayer was formed. The cell layer was scraped in a straight line using a 200μl pipette tip, keeping the tip perpendicular to the bottom of the well. After the scratch, a gentle wash was given to remove detached cells. The cells were given respective treatments, and images were taken under a microscope with 4X objective at 0hr, 24hrs, and 48hrs.

### 2.5 Invasion assay

HCT-116 cells were seeded at a seeding density of 0.01x10^6^ cells/well into the upper chamber of trans well inserts (Corning, #3422) precoated with 0.2% Gelatin (Sigma, #G2500) in serum-free media. The lower chamber was loaded with complete DMEM with 10% FBS, sufficient to touch the bottom of the inserts. 48 hours incubation post-treatment with TGF-β (10 ng/ml) ± WM (0.5μM) at 37°C, inserts were gently washed in 1X PBS, and the cells remaining in the upper chamber were removed with a cotton swab. The cells that invaded the other side of the insert membrane were fixed with 4% Paraformaldehyde (PFA) for 15 minutes, followed by permeabilization with 100% methanol for 20-30 minutes. The cells were stained with crystal violet for 20 minutes, and images were taken in five different fields at 10X objective magnification. The average number of cells per field was recorded. The stains taken up by the cells were dissolved in 10% SDS, and absorbance was measured at 540 nm.

### 2.6 Western blotting

0.1 x 10^6^ cells/well of HCT-116 were seeded in a 12-well cell culture plate and allowed to adhere properly. The cells were then pre-treated with 0.5μM WM followed by exposure to 10ng/ml TGF-β for 48 hours. The cells were pelleted in 1X PBS and lysed using ice-cold RIPA buffer with protease and phosphatase inhibitors on ice for 30 minutes. The whole cell lysate was centrifuged at 12,000 rpm for 15 minutes at 4°C, and the supernatant was collected. The protein concentration of the lysate was quantified using the Bradford assay, and an equal concentration of sample proteins was subjected to SDS-PAGE and transferred onto 0.45μm PVDF membrane (Millipore, Immobilon IPVH00010) by using the semidry Western Blotting method (BioRad). The non-specific binding was blocked with 5% skimmed milk in 1X TBST for 1 hour. After thorough washing, blots were probed with primary antibodies of N-CADHERIN (CST, #13116S), SNAIL (CST, #5879S), SLUG (CST, #9585S), Total SMAD2/3 (CST, #8685), phospho-SMAD2/3 (CST, #8828), PD-L1 (CST, #13684), and β-ACTIN (Sigma Aldrich, A3854) overnight at 4°C and HRP conjugated anti-rabbit IgG secondary antibody (Jackson Immuno Research Laboratories, #111-035003) for 4-5 hrs in 4°C. After thorough washing the protein signals were visualized using enhanced chemiluminescence (ECL) kit (Bio-Rad, #1705061).

### 2.7 Immunofluorescence

Briefly, 0.025 X 10^6^ HCT-116 cells were seeded onto the coverslip and allowed to adhere overnight. The cells were pre-treated with 0.5μM of WM 60 min before exposure to TGF-β for 48 hours. The cells were fixed with 3.7% PFA for 10 mins at room temperature and probed with a primary antibody phospho-SMAD2/3 overnight at 4°C or at room temperature for 1 hour. This was followed by probing with fluorescent-tagged secondary antibody (CST, # 4412) in the dark for one hour at room temperature. 1μg/ml of DAPI was used to stain the nucleus. After thorough washing with 1XPBS to remove the excessive stain, the coverslips were mounted onto glass slides and observed under a confocal microscope at 63X objective. MFI was calculated with the help of ImageJ.

### 2.8 Statistical analysis

The graphical data are expressed as mean ± SD of 3 or more independent experiment sets. Statistical tests were performed using GraphPad Prism Software version 8.0.2. Comparisons of the observed data were analyzed by Student *t* test distribution and one-way ANOVA.

## 3 Results

### 3.1 WM exhibits cytotoxicity to cancer cells

**Figure 1:**
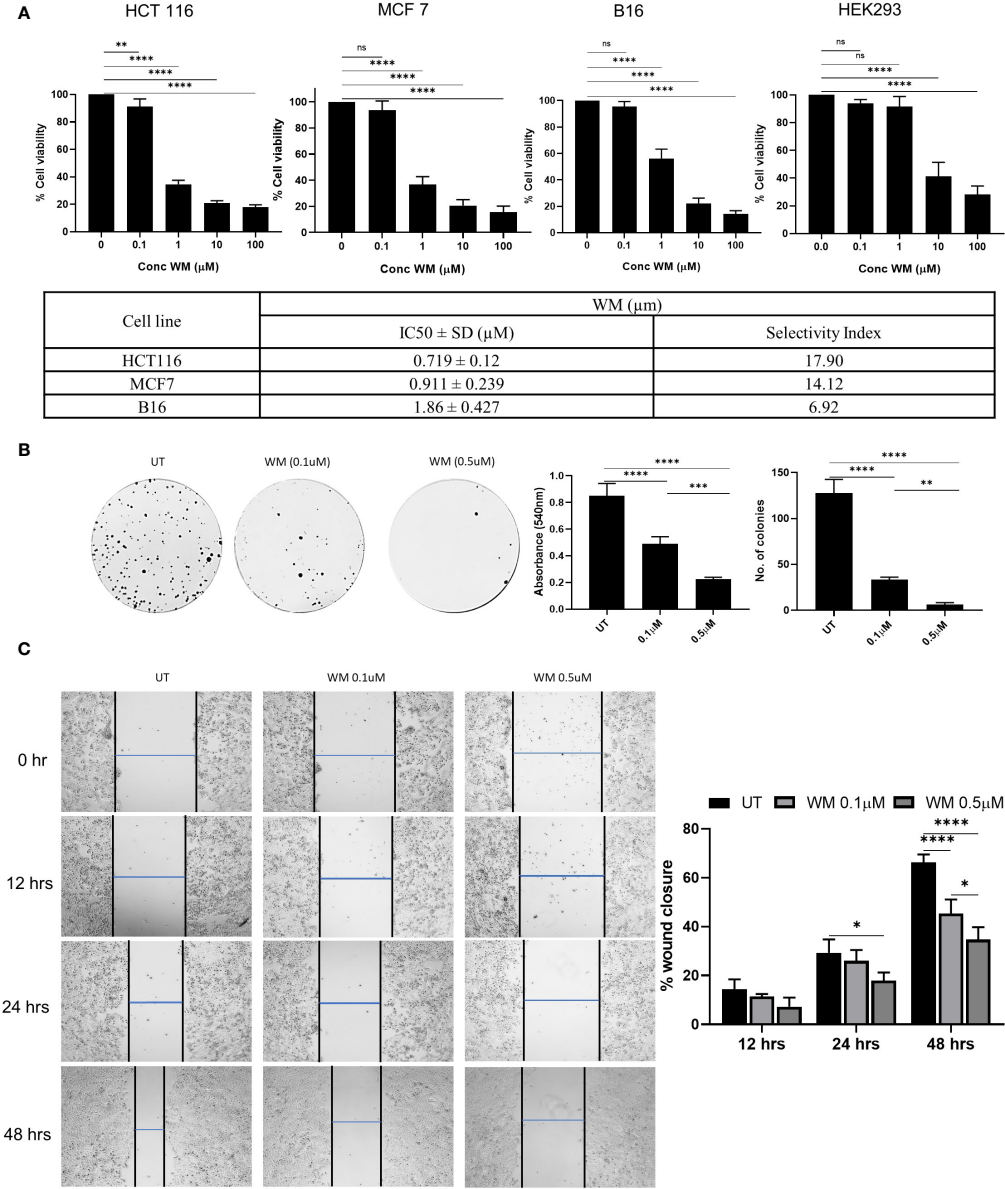
Effect of WM on HCT-116 colon adenocarcinoma cell line. **(A)** Dose-dependent inhibition of the growth of HCT-116 cell along with MCF-7 & B16 melanoma and the selectivity index calculated based on HEK293 (72 hrs). **(B)** Impact of WM (0.1μM, 1 μM) on the colony-forming ability of HCT-116 cells assessed by crystal violet staining on day 9 post treatment. **(C)** Representative images of wound healing assay performed in HCT-116 cells in the presence of WM (0.1μM, 1 μM) at 4X objective. Graphical representation of percentage of Wound healing in the presence of WM. Experiments were performed in triplicate. Data represented as mean ± SD. Comparisons of the observed data were analyzed using one-way ANOVA. *p < 0.05, **p<0.01, ***p<0.001, ****p<0.0001 versus the untreated.

In our study, we tested the cytotoxicity effect of WM on multiple cell lines at varying concentrations. We observed that WM exhibited a significant impact on the viability of HCT-116, MCF-7, and B-16 melanoma cells when compared to the non-cancerous cell line HEK-293. Among these, WM had the highest impact on HCT-116 with an IC_50_ value of 0.719 ± 0.12μM and a higher selectivity index (**Figure 1A**). To analyze further the impact of WM on cancer cell progression at lower concentrations, colony forming assay and wound healing assay were performed. As shown in **Figures 1B, C**, WM treatment significantly reduced the colony-forming ability and migration of HCT-116 in a dose-dependent manner. These observations suggest that WM exhibits anticancer potential even at sub-micromolar (<1μM) concentration with a notable selectivity index.

### 3.2 WM blocks TGF-β induced colony formation, migration, and EMT in HCT-116 cells

**Figure 2:**
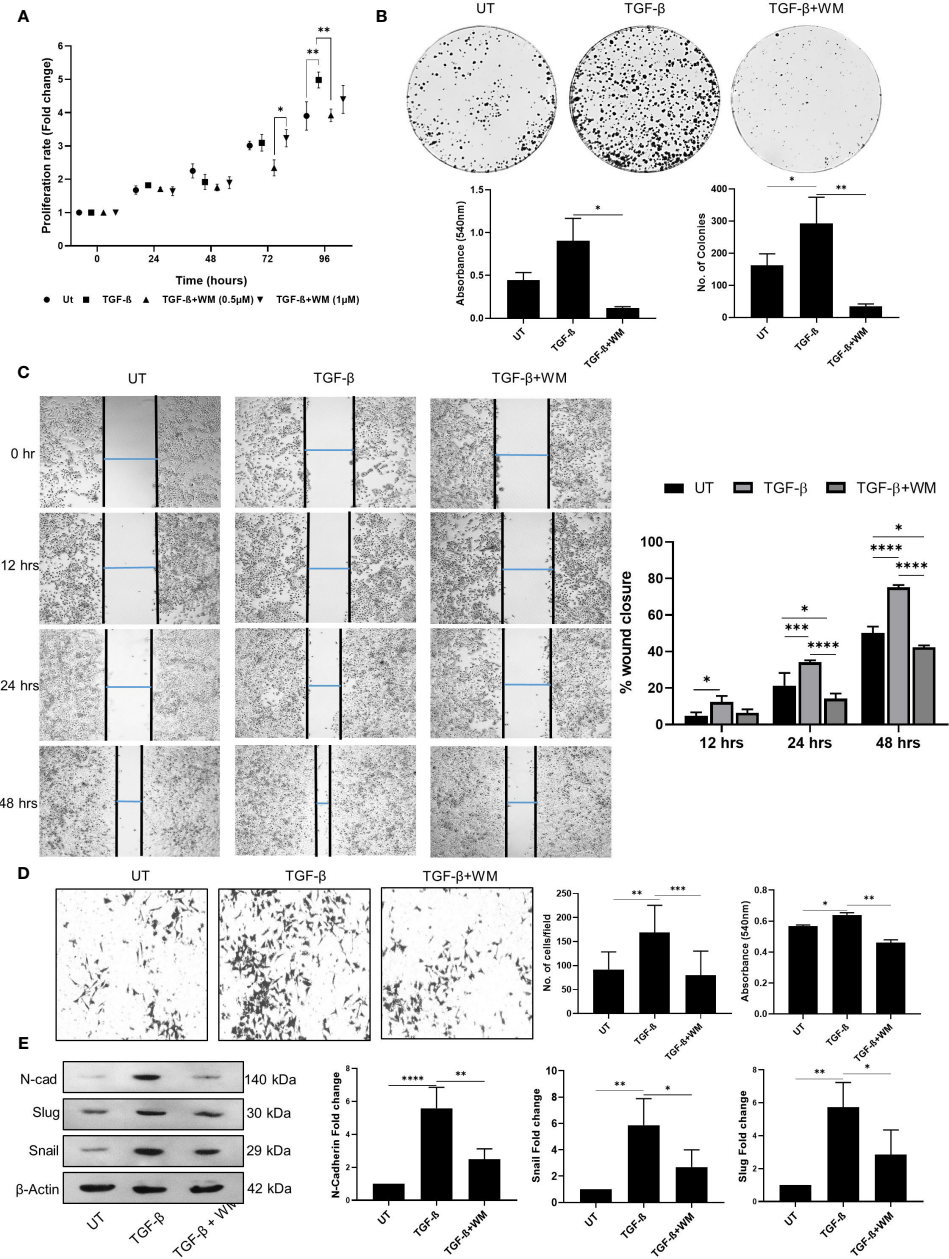
TGF-β induced cancer cell phenotype in HCT-116 cells is inhibited by Withametelin (WM). **(A)** MTT assay-based quantification of the growth of HCT-116 cell cultured in combination with TGF-β (10 ng/ml) ± WM (0.5μM). **(B)** Impact of WM on the TGF-β induced colony forming ability of HCT-116 cells assessed by crystal violet staining on day 9 post treatment. **(C)** Representative images of wound healing assay performed in HCT-116 cells in the presence of TGF-β ± WM (4X objective) and graphical representation of percentage of wound healing in the presence of TGF-β ± WM. E. **(D)** Invasion assay in HCT-116 cells in the presence of TGF-β ± WM (10X objective). WM inhibits TGF-β induced EMT marker expression in HCT-116 cells. **(E)** Western blot for N-CADHERIN, SNAIL & SLUG in HCT-116 cells treated with TGF-β ± WM for 48 hrs and densitometry quantification of western blots with respect to β-Actin. Experiments were performed in triplicate Data represented as mean ± SD. Comparisons of the observed data were analyzed using one-way ANOVA. *p<0.05, **p<0.01, ***p<0.001, ****p<0.0001.

Cytokines and chemokines in the tumor microenvironment play a critical role as mediators regulating a broad range of processes involved in tumor progression. Among these, TGF-β in the tumor microenvironment can potentially inhibit tumor initiation at an early stage, whereas it can promote late-stage cancer progression by accelerating proliferation, invasion, and metastasis (25). Selective targeting of TGF-β receptor and signaling has been explored for anticancer therapeutics with limited side effects (26). In this aspect, our study tested the ability of WM to regulate TGF-β induced cancer cell proliferation and EMT. We observed a marked increase in cell proliferation when treated with TGF-β at 72 hours and 96 hours incubation time points. However, this stimulatory effect of TGF-β was significantly reduced when the cells were pre-treated with 0.5μM of WM (**Figure 2A**). A similar pattern was observed in the colony-forming ability, and rate of migration of HCT-116 cells (**Figures 2B, C**). Additionally, the invasive property imparted by the presence of TGF-β is significantly reduced by WM as assessed by following trans-well insert assay (**Figure 2D**). Lastly, the causality of TGF-β on HCT-116 cells is tested in the presence of WM, and we found that mesenchymal markers such as N-CADHERIN and transcription factors such as SNAIL and SLUG are upregulated as part of EMT. We observed that the heightened expression of mesenchymal markers induced by TGF-β is inhibited by WM (**Figure 2E**). These observations suggest that WM could modulate not only the basal level progression of cancer cells but also reverse the pro-tumorigenic effect originating from TGF-β in HCT-116 cells.

### 3.3 TGF-β phosphorylation of SMAD2/3 is reduced in the presence of WM

**Figure 3:**
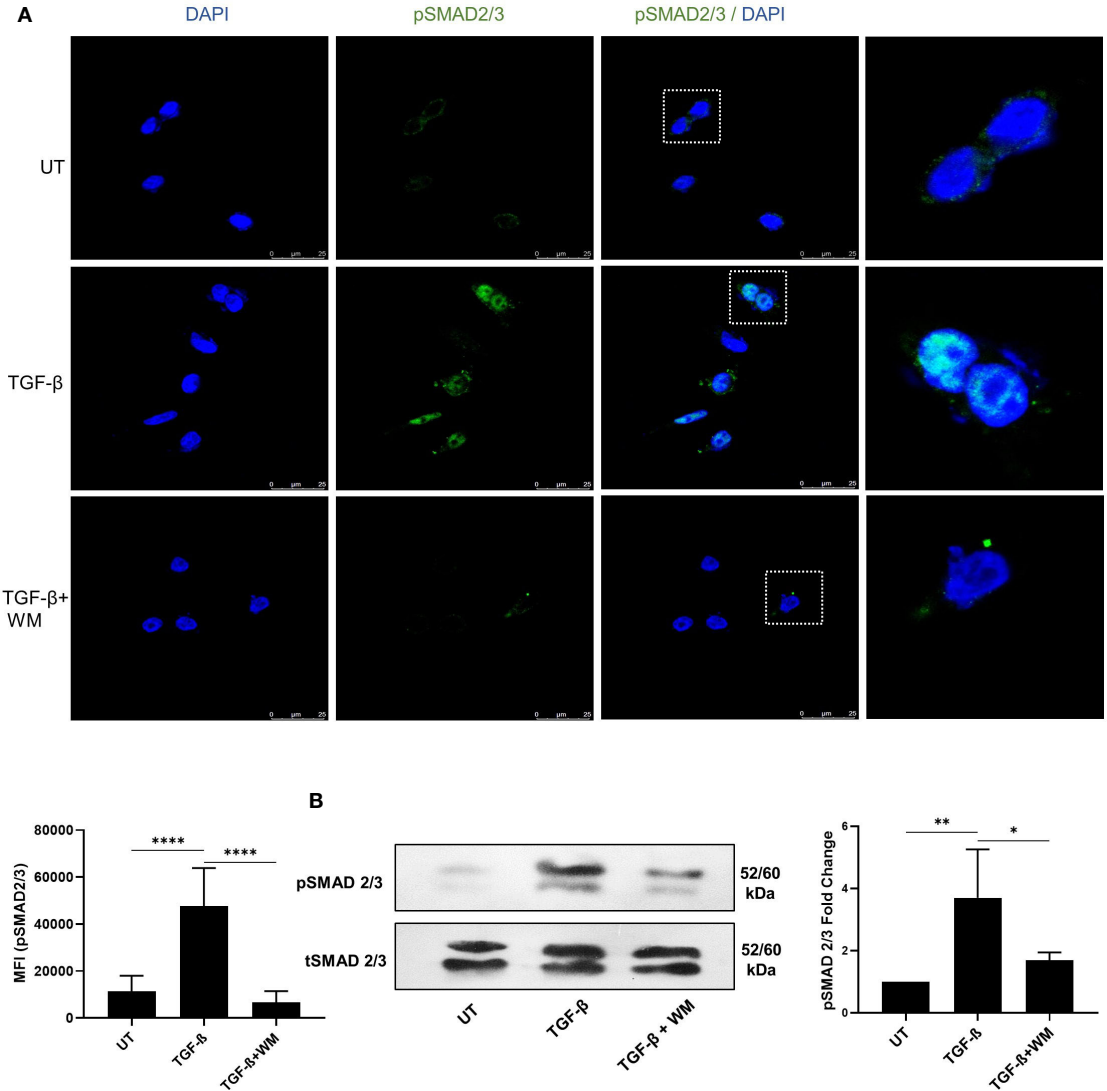
WM blocks TGF-β induced phosphorylation of SMAD2/3 in HCT116. **(A)** fluorescence-based quantification (63x objective) of pSMAD2/3 in HCT-116 after 48 hrs of incubation with TGF-β (10 ng/ml) ± WM (0.5μM). **(B)** western blot analysis and densitometry of pSMAD2/3 in treated HCT-116 cells. Experiments were performed in triplicate. triplicate Data represented as mean ± SD. Comparisons of the observed data were analyzed using one-way ANOVA. *p<0.05, **p<0.01, ***p<0.001, ****p<0.0001.

Mechanistically, it is widely accepted that classical TGF-β signaling involves binding of TGF-β to its receptors (TGF-βRI, TGF-βRII, TGF-βRIII) and activates Smad2 and Smad3. The activation is followed by the phosphorylation of SMAD2/3, which partners with SMAD4 and is translocated into the nucleus, where the SMAD complex acts as a transcription factor controlling the expression of various target genes, favoring EMT (27). Our experiments showed that WM hampered the phosphorylation of SMAD 2/3 (**Figure 3A**). The expression was analyzed based on the immunofluorescence assay, which clearly distinguished the prevalence of pSMAD2/3 in TGF-β treated cells compared to WM-pre-treated cells. Additionally, a similar outcome is seen in total cell lysates analyzed for pSMAD2/3 in the western blot (**Figure 3B**). These observations established that WM regulates TGF-β signaling by influencing SMAD2/3 phosphorylation.

### 3.4 WM regulates TGF-β induced expression of immune checkpoint inhibitor, PD-L1, on HCT-116 cells

**Figure 4:**
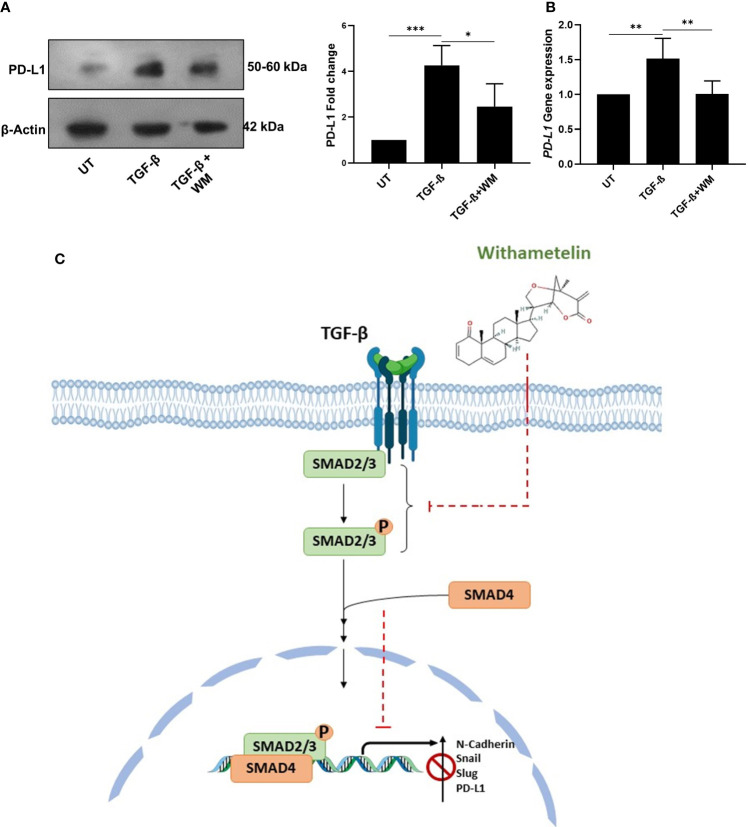
WM blocks TGF-β induced immune checkpoint inhibitor PD-L1 in HCT-116 cells. **(A)** western blot analysis and densitometry PD-L1 expression in HCT-116 cell lysates after 48 hrs treatment with TGF-β (10 ng/ml) ± WM (0.5μM). **(B)** qPCR analysis of PD-L1 gene expression in HCT-116 cell lysates treated with 48 hrs of TGF-β ± WM. Experiments were performed in triplicate. **(C)** Schematic representation of WM intercepting TGF-β induced EMT & PD-L1 in colon cancer cells. Data represented as mean ± SD. Comparisons of the observed data were analyzed using one-way ANOVA. *p<0.05, **p<0.01, ***p<0.001.

In the tumor microenvironment, elevated level of TGF-β regulates the activation of various immune cells, leading to inhibition of immune surveillance. Accumulating data suggests that TGF-β mediated immune evasion is also associated with its ability to regulate immune checkpoint inhibitors, such as PD-L1 (28). Therefore, we further focused on understanding the influence of TGF-β on PD-L1 expression in colon cancer cells and determining the effect of WM. Towards this, HCT-116 cells were exposed to TGF-β with and without WM, and western blot and q-RT PCR were performed to analyze the protein level expression of PD-L1. As shown in **Figures 4A, B** WM significantly downregulated the expression of TGF-β induced PD-L1. These results suggest that WM may intercept TGF-β signaling and regulate PD-L1 expression in colon cancer cells.

## 4 Discussion

TGF-β signaling contributes to maintaining the gastrointestinal epithelial cells, immune cells, and stromal compartments (29). However, the altered activity of TGF-β signaling has been reported to promote a diverse range of disease conditions, including Inflammatory Bowel Disease (IBD) and, subsequently, tumor initiation, progression, and metastasis (30–32). The dichotomous role of TGF-β as both a tumor suppressor and promoter has long been a barrier to developing effective molecular targeted therapies (33). However, clinical studies have shown that severe prognoses in colon cancers are accompanied by high levels of TGF-β in tumor tissues, highlighting its significant role in colon cancer progression (20). Hence, predominantly targeting the TGF-β signaling pathway can be a potential therapeutic strategy for IBD and colon cancers.

WM, a biologically active Withanolide isolated from the leaves of *Datura innoxa*, exhibits anti-depressant, antioxidant, anti-inflammatory, and anticancer activity (15). WM contributed to neuroprotection through the modulation of oxidative stress and neuroinflammation induced by TLR4/IκB-α/NF-κB/AP-1 signaling in the Central nervous system (34). WM has also been shown to alleviate diabetic neuropathic pain behavior in hyperglycemic rats by inhibiting MAPK/NF-κB signaling in the spinal cord (35). However, the functional significance of WM in CRC remains largely unexplored. Our study focused on exploring the anticancer potential of WM in the presence of TGF-β signaling *in vitro*. The cytotoxicity of WM towards colon cancer cell line, HCT-116, was determined using an MTT assay. We observed that WM imposes an anti-proliferative effect on HCT-116 cells at an IC_50_ value of 0.719 ± 0.12μM. The significant reduction in cell migration and the colony-forming ability of the cells at a very low dosage (**Figure 1**) pointed toward the promising efficacy of WM as an anticancer agent.

We further investigated the cellular response to TGF-β in promoting EMT in the presence of WM. The TGF-β induced proliferation, migration, invasiveness, and colony formation of cancer cells were significantly inhibited in WM-treated cells. The EMT is characterized by loss of cell adhesion and upregulation of mesenchymal markers. As mesenchymal markers, we examined N-CADHERIN, SNAIL, and SLUG. WM effectively inhibited EMT by downregulating the expression of mesenchymal markers induced by TGF-β (**Figure 2**). This inhibition could occur due to varying mechanisms, including the regulation of the TGF-β signaling pathway. Following the activation of the TGF-β type I receptor, there is a rapid phosphorylation and activation of Smad transcription factors (Smad 2 and 3). Activated Smads translocate into the nucleus and initiate gene expression (36). WM successfully diminished the phosphorylation of Smad2/3 and associated gene expression (**Figure 3**). The results signify that WM inhibits TGF-β induced EMT by inhibiting phosphorylation of SMAD2/3 complex.

Recent studies suggest that TGF-β mediated upregulation of PD-L1 is selectively through the direct binding of SMAD3 to the Smad-binding elements (SBEs) located upstream of the Pdcd-1 transcription start site (37). Despite multiples studies highlighting the association between TGF-β1 signaling and high PD-1 expression, the exact molecular mechanism still remains undetermined.

One of the landmark events in cancer immunotherapy is the discovery of immune checkpoints, such as PD-L1, and the development of monoclonal antibody-based therapeutics (38). Antibody mediated targeting of PD-L1 inhibits its interaction with PD1, thereby potentiating the proliferation of T cells and re-establishment of immune response (38). Despite showing sustained antitumor effects across multiple cancers, the low response rate remains a major drawback for anti-PD-1/PD-L1 therapies (39). Studies have reported TGF-β as a determinant for anti-PD-1/PD-L1 therapies (40). Many combinatorial therapies targeting TGF-β-signaling and anti-PD-L1 therapy are under clinical investigation. This includes BiTP antibody targeting human TGF-β and PD-L1 (41). A recent study showed that dual inhibition of TGF-β and PD1/PD-L1 with combination of ALK5i and anti-PD-L1 antibody, caused a significant increase in the frequency of CD3^+^T cells and enhanced the infiltration of CD8+ T cells in KPN liver tumor metastasis, a metastatic mouse model for CMS4 subtype of CRC (42). But, simultaneous inhibition of TGFβR1 pathway and PD-L1 in isolation contributed towards upregulation of a metabolic gene expression signature that favored cancer cell proliferation suggesting a need for alternative strategies to control cancer metastasis. In this context, single-molecule treatment targeting TGF-β induced PD-L1 in its downstream without effecting the non-canonical arm of the signaling pathway may have the potential for an optimal suppression of cancer progression, a benefit not provided by combining independent therapies. Towards this, WM-treated HCT-116 cells showed reduced PD-L1 expression, which may make them more susceptible to immune surveillance (**Figure 4**). However the exact molecular mechanism of WM inhibiting TGF-β signaling pathway still remains to be explored. Finally, our findings provide insight into the anticancer and immunomodulatory potential of WM. We suggest that WM inhibits TGF-β induced EMT in colon cancer by blocking TGF-β induced expression of PD-L1 in cancer cells (**Figure 4C**) which may likely prevent tumor evasion from the immune system, possibly implicating its utility as an anticancer agent.

## Data availability statement

The raw data supporting the conclusions of this article will be made available by the authors, without undue reservation.

## Ethics statement

Ethical approval was not required for the studies on humans in accordance with the local legislation and institutional requirements because only commercially available established cell lines were used.

## Author contributions

AF: Data curation, Investigation, Writing – original draft, Writing – review & editing. MF: Resources, Writing – review & editing. SB: Conceptualization, Resources, Writing – review & editing. TJ: Conceptualization, Data curation, Funding acquisition, Investigation, Project administration, Supervision, Writing – original draft, Writing – review & editing.
